# Data on Rad51 amino acid sequences from higher and lower eukaryotic model organisms and parasites

**DOI:** 10.1016/j.dib.2016.12.002

**Published:** 2016-12-08

**Authors:** Andrew A. Kelso, Steven D. Goodson, Lesly A. Temesvari, Michael G. Sehorn

**Affiliations:** a Department of Genetics and Biochemistry, Clemson University, Clemson 29634, SC, USA; b Eukaryotic Pathogens Innovation Center, Clemson University, Clemson 29634, SC, USA; c Department of Biological Sciences, Clemson University, Clemson 29634, SC, USA; d Clemson University School of Health Research, Clemson 29634, SC, USA; e Center for Optical Materials Science and Engineering Technologies, Clemson University, Clemson 29634, SC, USA

**Keywords:** Rad51, Recombinase, Eukaryotic pathogen

## Abstract

This paper contains data related to the research article titled “Characterization of the recombination activities of the *Entamoeba histolytica* Rad51 recombinase” (Kelso et al., in press) [1]. The known and putative amino acid sequence of Rad51, the central enzyme of homologous recombination, from nineteen different higher and lower eukaryotic organisms was analyzed. Here, we show amino acid conservation using a multiple sequence alignment, overall sequence identities using a percent identity matrix, and the evolutionary relationship between organisms using a neighbor-joining tree.

**Specifications Table**

**Table:** 

Subject area	*Biology*
More specific subject area	*Genetics, Biochemistry, and Molecular Biology*
Type of data	*Figures and Table*
How data was acquired	*Bioinformatic analysis*
Data format	*Raw and analyzed*
Experimental factors	*Reference sequences were downloaded from UniProt.*
Experimental features	*Analysis was performed using MUSCLE and Clustal2.1*
Data source location	*Clemson University, Clemson, SC, USA*
Data accessibility	*Data are available in this article*

**Value of the data**

• From the presented sequence alignment data of 19 different Rad51 orthologs, highly conserved amino acids (including complete positive and negative conservation) can be identified for mutagenic studies on Rad51 to determine functional conservation.
• The Rad51 sequence identity shows the relatedness of Rad51 between many organisms, useful for future genetic and biochemical studies in the presented organisms and in organisms in which Rad51 is uncharacterized.
• The neighbor-joining tree data shed light on the phylogenic relationship between Rad51 from several higher eukaryotic organisms and eukaryotic pathogens. This is valuable for studies comparing the phylogeny of other highly conserved homologous recombination genes.

## 1 Data

**Fig. 1:**
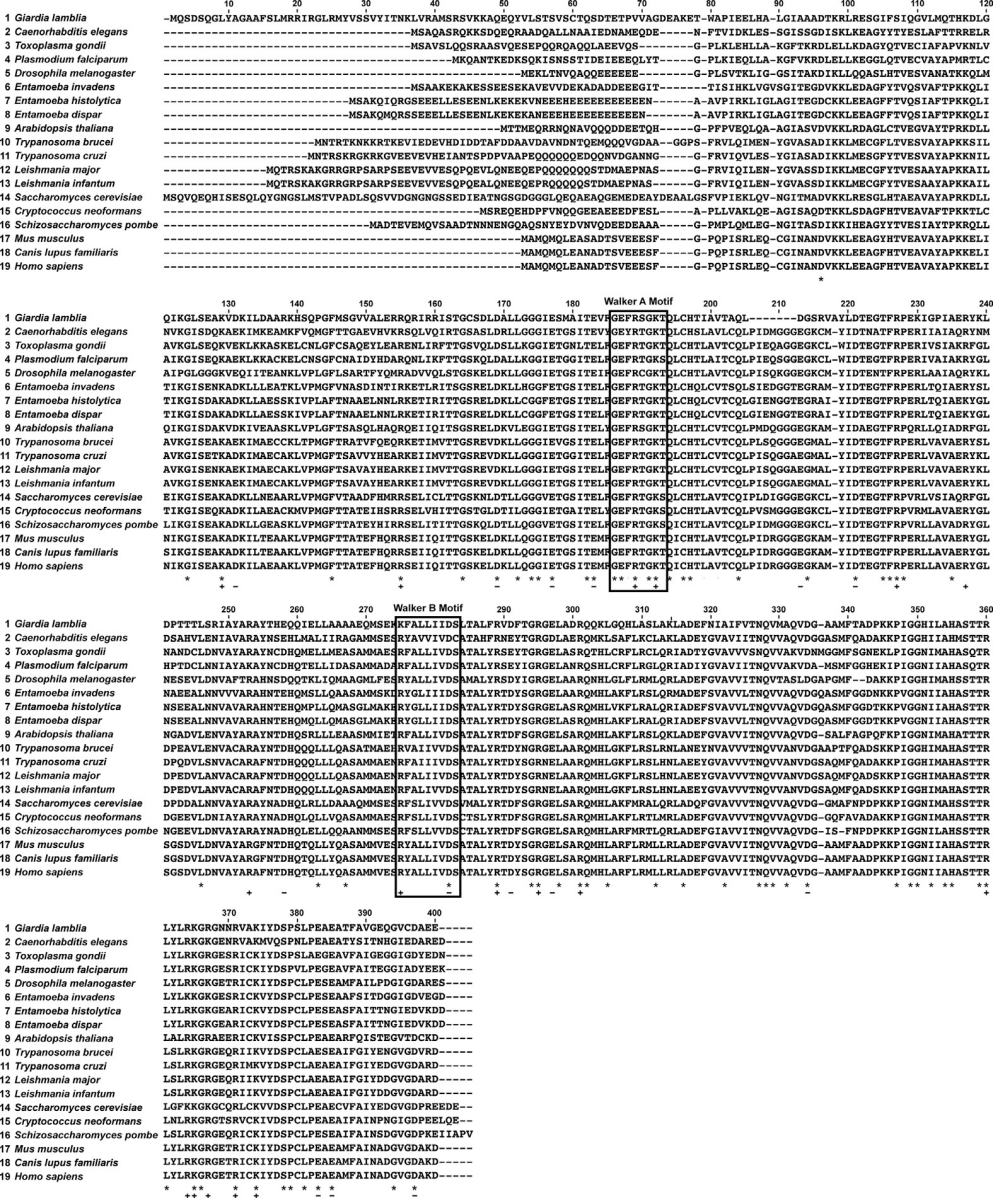
Rad51 protein sequence alignment. A multiple sequence alignment of Rad51 protein sequences was performed using MUSCLE. The boxes represent the highly conserved Walker A and Walker B motifs (as indicated). * indicates complete conservation of the amino acid, − indicates all negative amino acids, and + indicates all positive amino acids.

**Fig. 2:**
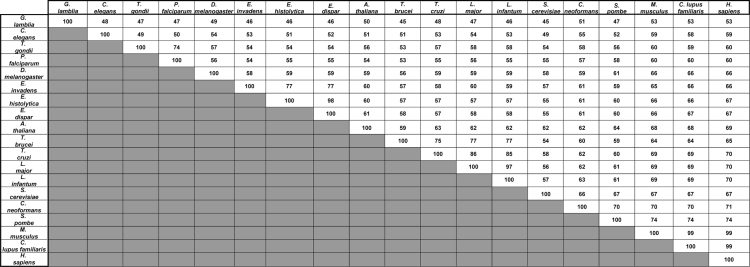
Percent identity matrix of Rad51 protein sequences. The data were retrieved from Clustal2.1.

**Fig. 3:**
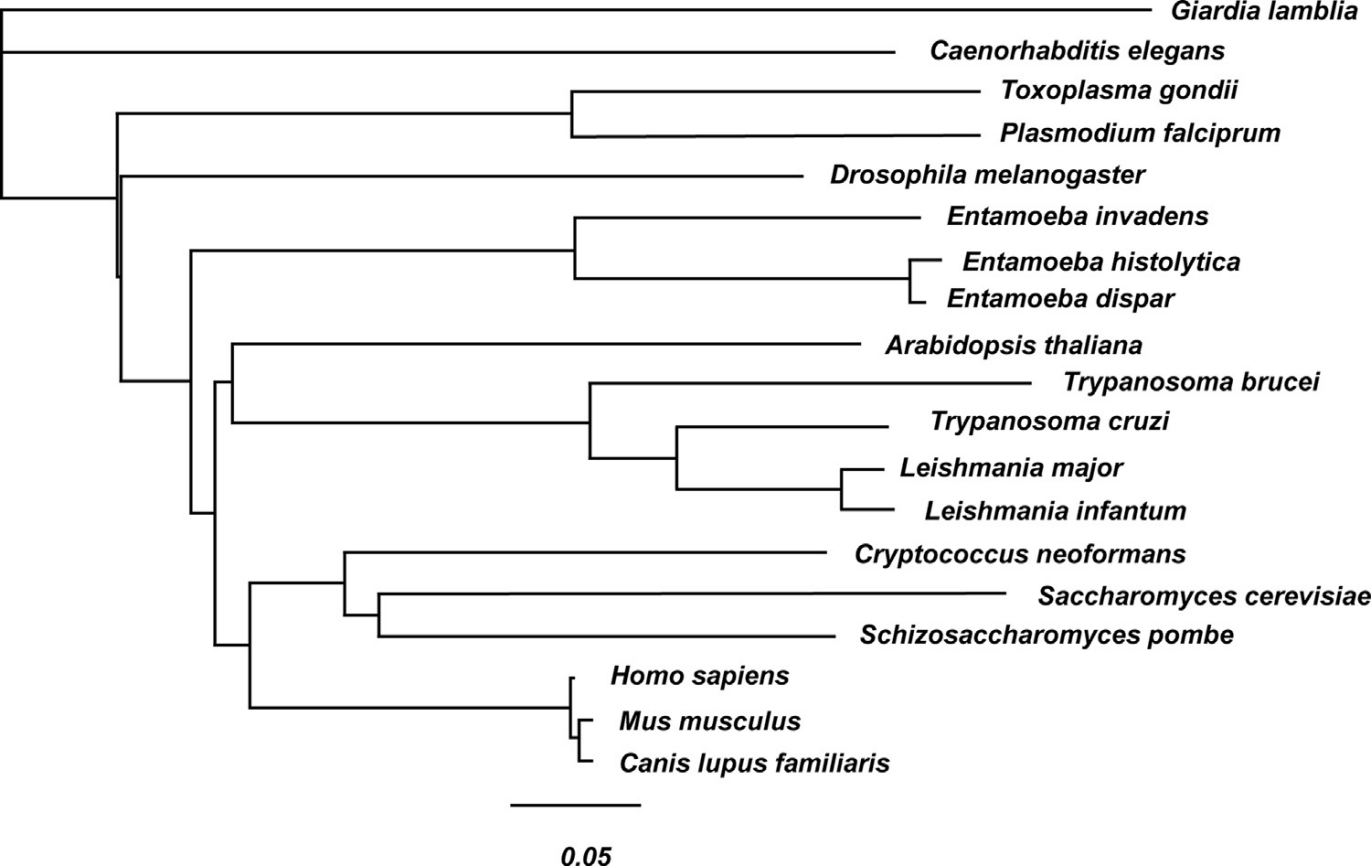
Neighbor-joining tree based on Rad51 protein sequences. The data were retrieved from Clustal2.1, and the tree was constructed using MUSCLE and edited using Geneious 9.1.5.

The data described, include supporting information on sequence conservation and identity of Rad51 for the analysis by Kelso et al., in press [1]. A Rad51 amino acid sequence alignment from nineteen different vertebrate and invertebrate organisms is shown. In the alignment, the highly conserved Walker A and B motifs [2], [3] are highlighted, along with amino acids that are completely conserved, and completely positive or negative (Fig. 1). Also, to emphasize the relatedness of the Rad51 amino acid sequence from each eukaryotic organism, a percent identity matrix is presented (Fig. 2). Lastly, for phylogenetic analysis, a neighbor-joining tree is presented showing the evolutionary relationship of Rad51 (Fig. 3). A comparison of *Entamoeba histolytica* Rad51 to the other species was analyzed in the previously mentioned article [1] and by Lopez-Casamichana et al., 2008 [4].

## 2 Experimental design, materials and methods

Rad51 reference sequences for each organism were downloaded from UniProt [5] (http://www.uniprot.org/). Rad51 UniProt sequence identifiers and the corresponding GenBank accession numbers for each of the represented species can be found in Table 1. Using these amino acid sequences, a multiple sequence alignment was performed using MUSCLE (3.8) [6], [7] (www.ebi.ac.uk/Tools/msa/muscle/). A percent identity matrix was prepared using data retrieved from Clustal2.1 [8] (www.ebi.ac.uk/). A neighbor-joining tree was assembled from the multiple sequence alignment data using the Jukes-Cantor genetic distance model and edited using Geneious 9.1.5 (www.geneious.com).

## Figures and Tables

**Table 1:** Species, identifiers, and accession numbers of Rad51 sequences.

**Species**	**UniProt identifier**	**GenBank accession number**
*Entamoeba histolytica*	Q86C17	AAP35107.1
*Homo sapiens*	Q06609	CAG38796.1
*Saccharomyces cerevisiae*	P25454	CAA45563.1
*Schizosaccharomyces pombe*	P36601	CAA80879.1
*Mus musculus*	Q08297	NP_035364.1
*Arabidopsis thaliana*	P94102	OAO95923.1
*Drosophila melanogaster*	Q27297	BAA04580.1
*Caenorhabditis elegans*	G5EGG8	AAD10194.1
*Canis lupus familiaris*	Q8MKI8	BAB91246.1
*Entamoeba dispar*	B0EJ35	EDR25461.1
*Entamoeba invadens*	A0A0A1U2S7	ELP88329.1
*Trypanosoma brucei*	Q384K0	AAD51713.1
*Trypanosoma cruzi*	Q4CYE3	AAZ94621.1
*Leishmania major*	O61127	AAC16334.1
*Leishmania infantum*	A4I3C9	XP_001470091.1
*Toxoplasma gondii*	I6XGP4	AFN55127.1
*Plasmodium falciparum*	Q8IIS8	XP_001347762.2
*Giardia lamblia*	V6U507	XP_001709425.1
*Cryptococcus neoformans*	Q5KNC3	XP_012046913.1
